# The Management of Glaucoma: Structure and Function

**DOI:** 10.1155/2018/4682586

**Published:** 2018-04-22

**Authors:** Kazuyuki Hirooka, Tomomi Higashide, Jin Wook Jeoung

**Affiliations:** ^1^Department of Ophthalmology, Kagawa University Faculty of Medicine, Kagawa 761-0793, Japan; ^2^Department of Ophthalmology, Kanazawa University Graduate School of Medical Science, Kanazawa 920-8641, Japan; ^3^Department of Ophthalmology, Seoul National University College of Medicine, Seoul 03080, Republic of Korea

Since the introduction of optical coherence tomography (OCT), this procedure has transformed both the diagnosis and the measurement of the treatment efficacy in glaucoma patients. Furthermore, OCT has become one of the most common imaging modes for evaluations of the optic nerve head (ONH) [1], the retinal nerve fiber layer (RNFL) [2], and the macular thickness [3]. When diagnosing and monitoring glaucoma patients, both structural and functional tests are necessary. Thanks to advances in OCT imaging, such as swept-source OCT and enhanced depth imaging OCT, this has made it possible to perform in vivo imaging of the ONH deep tissues [4, 5]. In addition, it is now possible to differentiate healthy eyes, suspected glaucoma, and glaucomatous eyes of varying severities based on the quantitative assessments of the microcirculation in the ONH and peripapillary region. Furthermore, the diagnostic accuracy that is seen with this technology has been demonstrated to be similar to that seen for both the visual fields [6] and the RNFL thickness [7].

The purpose of this special issue is to present several new approaches that use OCT in the management of glaucoma patients. For this special edition, we received nine submissions in total, and based on these valuable review reports, we have accepted seven original high-quality research articles for publication within this issue. The papers contained within cover a variety of topics and approaches, ranging from the observation of the goniostructure to examinations of the bleb wall after trabeculectomy, along with investigations of the RNFL and macular ganglion cell-inner plexiform layer thickness or optic disc, impact adherence, and the morphology of the scotoma. Moreover, the novel and innovative approaches discussed in these articles may help to stimulate further research into the management of glaucoma patients. The team of guest editors chosen to select the papers for inclusion in this special issue believes that the results included here reflect recent and current trends in this exciting research field, in addition to outlining new ideas for further studies into the management of glaucoma patients.
